# Complete Genome Sequences of Eight *Helicobacter pylori* Strains with Different Virulence Factor Genotypes and Methylation Profiles, Isolated from Patients with Diverse Gastrointestinal Diseases on Okinawa Island, Japan, Determined Using PacBio Single-Molecule Real-Time Technology

**DOI:** 10.1128/genomeA.00286-14

**Published:** 2014-04-17

**Authors:** Kazuhito Satou, Akino Shiroma, Kuniko Teruya, Makiko Shimoji, Kazuma Nakano, Ayaka Juan, Hinako Tamotsu, Yasunobu Terabayashi, Misako Aoyama, Morimi Teruya, Rumiko Suzuki, Miyuki Matsuda, Akihiro Sekine, Nagisa Kinjo, Fukunori Kinjo, Yoshio Yamaoka, Takashi Hirano

**Affiliations:** a Okinawa Institute of Advanced Sciences, Uruma, Japan; b Okinawa Industrial Technology Center, Uruma, Japan; c Department of Environmental and Preventive Medicine, Oita University Faculty of Medicine, Yufu, Japan; d Pharmacogenomics Project, Kyoto University Graduate School of Medicine, Kyoto, Japan; e Department of Endoscopy, University Hospital, University of the Ryukyus, Nishihara, Japan; f Department of Medicine–Gastroenterology, Baylor College of Medicine and Michael E. DeBakey Veterans Affairs Medical Center, Houston, Texas, USA

## Abstract

We report the complete genome sequences of eight *Helicobacter pylori* strains isolated from patients with gastrointestinal diseases in Okinawa, Japan. Whole-genome sequencing and DNA methylation detection were performed using the PacBio platform. *De novo* assembly determined a single, complete contig for each strain. Furthermore, methylation analysis identified virulence factor genotype-dependent motifs.

## GENOME ANNOUNCEMENT

*Helicobacter pylori* is a spiral-shaped, Gram-negative, microaerophilic bacterium that colonizes the stomach. Its genome consists of a circular chromosome with a size of around 1.6 Mb, an average G+C content of 39% (1), and high allelic diversity (2). Approximately half of the world’s population harbors the bacterium (3). Infection with *H. pylori* occurs worldwide, but the prevalence varies greatly among populations (4). The vast majority of infected patients are asymptomatic; however, *H. pylori* infection is linked with the development of certain gastrointestinal diseases (5).

Okinawa is the southernmost prefecture of Japan, with a population of approximately 1.4 million people. Although there is no significant difference in *H. pylori* prevalence between Okinawa (42% in 2004) and other areas in Japan (6, 7), the incidence of gastric cancer in Okinawa (6.0 deaths/100,000 population in 2012) is by far the lowest in Japan (10.5 deaths/100,000 population in 2012) [Center for Cancer Control and Information Services, National Cancer Center, Japan; http://ganjoho.jp/data/professional/statistics/odjrh3000000hwsa-att/pref_CancerSite_mortalityASR75(1995-2012).xls]. In our previous study, we determined the genotypes of *cagA* and *vacA* virulence factors using PCR and Sanger-based sequencing technology and revealed an association between *H. pylori* virulence factors and gastroduodenal diseases in Okinawa (8). In the present study, we performed whole-genome sequencing and DNA methylation detection for eight *H. pylori* Okinawa strains using PacBio single-molecule real-time (SMRT) sequencing technology (9) to gain broader insights into the virulence of *H. pylori* (10, 11).

Next-generation sequencing (NGS) technologies are widely used in genomics studies. However, due to their PCR-amplification bias and shortness of read lengths, they are inadequate to generate the finished genome assemblies of *H. pylori* strains because of the low G+C content and large numbers of repetitive regions in such strains (12). In contrast, the SMRT technology provides real-time analysis of biomolecules at single-molecule resolution. It achieves unbiased G+C coverage (13), extraordinarily long, multikilobase reads (14), and direct methylation sequencing (15).

*H. pylori* Okinawa strains examined in the present study had been previously isolated from patients with gastric atrophy, gastric ulcer, or duodenal ulcer (8). Whole-genome sequencing of eight *H. pylori* Okinawa strains was carried out using the PacBio RS (Pacific Biosciences, Menlo Park, CA) platform with a 10-kb insert library and XL/C2 chemistry. *De novo* assembly was conducted using the hierarchical genome assembly process (HGAP) workflow (16), including consensus polishing with Quiver. This workflow resulted in a single, complete contig for each genome. Annotation was added by the NCBI Prokaryotic Genome Annotation Pipeline (PGAP) (17). DNA methylation detection was also carried out using the kinetics data collected during the sequencing process. A total of 24 methylation motifs were found to be common to two or more strains. Interestingly, some motifs were associated with the genotypes of virulence factors. A summary of statistics for these eight *H. pylori* Okinawa strains is shown in Table 1. A detailed comparative analysis of these genomes will be included in a future publication.

**TABLE 1:** Summary of statistics for the eight *H. pylori* Okinawa strains

Strain name	Clinical presentation	Coverage depth^*a*^ (fold)	Genome length (bases)	G+C content (%)	No. of genes	No. of CDS^*b*^	Accession no.
Oki102	Atrophy	489	1,633,212	38.81	1,610	1,478	CP006820
Oki112	Atrophy	328	1,637,925	38.81	1,620	1,477	CP006821
Oki128	Atrophy	261	1,553,826	38.97	1,534	1,393	CP006822
Oki154	Duodenal ulcer	822	1,599,700	38.80	1,584	1,439	CP006823
Oki422	Atrophy	432	1,634,852	38.83	1,620	1,468	CP006824
Oki673	Gastric ulcer	193	1,595,058	38.82	1,587	1,437	CP006825
Oki828	Duodenal ulcer	434	1,600,345	38.80	1,590	1,445	CP006826
Oki898	Duodenal ulcer	613	1,634,875	38.83	1,615	1,488	CP006827

a Postfilter polymerase read bases.

b CDS, coding sequences.

### Nucleotide sequence accession numbers.

The complete genome sequences of all eight *H. pylori* Okinawa strains have been deposited in DDBJ/EMBL/GenBank under the accession numbers listed in Table 1.
